# Safety and Inner City Neighborhood Change: Student and Teacher Perspectives

**DOI:** 10.1177/00131245211004553

**Published:** 2021-05-22

**Authors:** Sejal Patel, Miad Ranjbar, Tawnya C. Cummins, Natalie M. Cummins

**Affiliations:** 1 Ryerson University, Toronto, ON, Canada

**Keywords:** safety, school redesign, neighborhood revitalization, inner city, mixed-income communities

## Abstract

The introduction of mixed-income communities in public housing neighborhoods is a common revitalization strategy in metropolitan areas in North America. This study investigates student and teacher perspectives on safety in a Canadian inner city and marginalized neighborhood undergoing revitalization, alongside the redesign of a local school. The displacement of families and students, tied to housing relocation and student school mobility, resulted in increased concern around bullying, school safety, and displacement of place-based familiarity and social bonds. While most students felt safe at school, they were acutely aware of community level violence, criminal and gang activity in the neighborhood, and racial stereotyping. Students were also generally skeptical that revitalization would address the root causes of violence. The findings support the importance of including children’s voices when planning, implementing, and evaluating policy initiatives that directly affect their lives.

## Introduction

Community safety is an important indicator of children’s well-being and healthy development (Ben-Arieh, McDonell, & Attar-Shwartz, 2009). Research suggests that exposure to violence in the community can negatively affect children’s mental health outcomes (Galster & Santiago, 2006), physical health (Leventhal & Brooks-Gunn, 2000), and academic performance (McCoy et al., 2013). In neighborhoods prone to high rates of violence, neighborhood revitalization, and transformation are frequently suggested to alter the social ecology of a community (Maton, 2000), and ultimately reduce the prevalence of violence. A prominent revitalization strategy employed in many metropolitan areas is the introduction of mixed-income communities in public housing neighborhoods that experience high levels of criminal activity (Tach, 2009). Despite an increase in the adoption of such neighborhood change strategies, children’s experiences during (and after) the implementation of revitalization initiatives are relatively underexplored. Given the significant negative effects of community violence on children’s social and psychological well-being (Ben-Arieh et al., 2009), it is imperative to investigate children’s perspectives on school and community safety in redesigned neighborhoods.

A meta-analysis of 114 studies on the effects of exposure to community violence found that witnessing or hearing about violent behavior in the community affects children’s mental health in terms of emotional regulation, traumatization, and fear for safety, although direct victimization more strongly predicts the development of symptomatology (Fowler et al., 2009). Neighborhood exposure to violence affects children’s social and emotional well-being (Leventhal & Brooks-Gunn, 2000), including their ability to form relationships and develop a sense of mastery over the environment and trust (Overstreet, 2000).

Through its negative effects on children’s psychological well-being, exposure to violence is thought to impair educational achievement (Leventhal & Brooks-Gunn, 2004), and may influence long-term functioning of schools (McCoy et al., 2013). Neighborhood crime can impact the school climate by altering social norms about the use of violence to solve interpersonal disputes and by increasing aggressive behavior (McCoy et al., 2013). Furthermore, the cumulative impact of neighborhood crime on children’s academic performance is stronger when the school is in a low socioeconomic neighborhood (Galster & Santiago, 2006; Leventhal & Brooks-Gunn, 2000).

Moving higher income residents into a low socioeconomic neighborhood has been found to be positively correlated with children’s educational achievement (Leventhal & Brooks-Gunn, 2004) and mental health (Formoso et al., 2010). Formoso et al. (2010) suggest the presence of higher income residents improve child outcomes through the addition of high-quality institutional resources and the presence of “role-models” and social support for children. The introduction of mixed-income communities is seen as a strategy to also offset the stigmatization and discrimination that residents in areas of concentrated poverty face (Dunn, 2012). Others argue that such social engineering initiatives may instead negatively affect the residents of the neighborhood by reducing their sense of community (August, 2014). As seen in the case of HOPE VI in the United States, where higher income families were moved into impoverished inner city areas, mixed-income neighborhood redevelopment does not address the core causes of stigmatization rooted in poverty, social inequality (August, 2014) and racial segregation (Kost, 2012). It is these factors that are thought to continuously weaken residents’ perceptions of safety, by driving racially marginalized and low-income youth into local drug and gang-related activities, where “at most, [neighborhood redevelopment] may alter the geography of street-level dealing, by shifting it to a different area” (August, 2014, p. 1329).

Neighborhood revitalization initiatives are also criticized for legitimizing gentrification and displacement, with many residents never returning to their newly redesigned mixed-income communities after the disruption and dislocation caused by redevelopment (Lipman, 2009). For instance, rather than feeling safer, former residents of public housing neighborhoods felt more vulnerable due to their disrupted and diminished social ties (Clampet-Lundquist, 2010). These emergent findings contradict the “broken windows” theory (Wilson & Kelling, 1982), which attributes residents’ feelings of safety and the prevalence of violence and/or neighborhood criminal activity to the presence of visible signs of disorder, such as deteriorating structures, graffiti, and accumulated garbage or debris. These visual cues are thought to attract criminal offenders who take them as indicative of residents’ indifference to what goes on in their neighborhood.

Recent research on neighborhood safety have found the existence and prevalence of social ties and social cohesion to be more potent contributors to residents’ feelings of safety and their perceived risk of victimization in the neighborhoods (e.g., Hwang, 2016; Lipman, 2009; Sampson & Raudenbush, 1999). For example, Thompson et al. (2013) found that during the early stages of redevelopment of a public housing neighborhood in Toronto, Canada, young adults’ experiences were affected by a number of unintended outcomes, including perceived heightened risk of victimization and general loss of social networks and support systems (Thompson et al., 2013). Further, Hwang (2016), in her work on gentrifying neighborhoods, points to a clear disconnect between longstanding and new residents in how they construct their neighborhood identities and boundaries, with the latter group’s narratives excluding areas of the neighborhood comprised mainly of longstanding residents. In fact, the ease of forming meaningful social ties between longstanding and new residents has been contested (Clampet-Lundquist, 2010; Tach, 2009), raising questions in the underlying logic of mixed-income revitalization efforts.

Lipman (2009) notes that within the process of mixed-income redevelopment, creating newer or improved schools helps entice higher income families to move into these neighborhoods. While it is suggested that student achievement increases in schools that have been physically renovated (Uline & Tschannen-Moran, 2008), creating new or improved schools does not necessarily help address the causes of violence within the school setting and across the community (Lipman, 2008). This may be a result of conflating the concepts of space and place in planning and executing revitalization initiatives where improvements in “space”—that is, the physical characteristics of a given location—are believed to lead to improvements in “place,” a term used to refer to the confluence of both the physical and the psychosocial qualities that give meaning to that location (e.g., Kim et al., 2013).

In terms of violence prevention and student safety within the physical boundaries of a school, it is suggested that violence, including physical fights and bullying, is a systemic and school-wide issue, involving more than the dyadic relationship between a perpetrator and a victim (Pepler, 2006). Other research supports systemic approaches, placing emphasis on the relationship between students and teachers in alleviating violence and bullying in schools (Cortes & Kochenderfer-Ladd, 2014). Interventions to reduce violence and ensure student safety that focus on the use of security measures have mixed success, perhaps because visible security measures remind students of the high potential for school violence (Perumean-Chaney & Sutton, 2013). Instead, some advocate for refining interventions based on the profiles of perpetrators and victims of school violence (Olweus, 1997; Pepler, 2006).

Discussions of school violence, bullying, and victimization tend to be predominantly psychological, and the resultant psychological profiling of “bullies and victims” is believed by many researchers to impede our understanding of the true nature of violence as having social and political underpinnings (Walton, 2005). Perceptions of incivilities across the neighborhood impact students’ perceptions of safety, and neighborhood violence often finds its way into the school context by altering social norms regarding dealing with conflict (McCoy et al., 2013). A meta-analysis of school-wide violence prevention approaches in the US and European nations found modest positive outcomes, mostly in relation to knowledge, attitudes, and self-perceptions, rather than actual reductions in the prevalence of bullying and physical violence (Merrell et al., 2008). The relationship between violence and safety, educational achievement, and well-being is complex, and may not be easily improved by social interventions such as neighborhood revitalization and mixed-income initiatives.

Unlike urban renewal efforts of the past, promising results of current revitalization initiatives in established urban neighborhoods requires some degree of input from the individuals affected by such programs (Mercier, 2003). While unintended social outcomes remain a possibility (Thompson et al., 2013), planning processes that involve the voices of a community’s residents can result in better outcomes (Walker & East, 2014) by helping to empower individuals who are involved in the process (Phillips et al., 2010). Multi-stakeholder discussions of safety issues within and across different communities is key (Ben-Arieh et al., 2009). Specifically, attending to student input is important because students, teachers, and parents often have starkly different views about safety (e.g., what actions count as unsafe, what places in the neighborhood are most associated with violence), and relying on data from one party may result in inappropriate conclusions (Ben-Arieh et al., 2009). Furthermore, as literature of school violence suggests, school climate is unique and students’ experiences are context-dependent, with students primarily affected by interpersonal violence, such as bullying (Rees, 2002). Finally, by drawing on contemporary theories and research in childhood sociology, children should be seen as experts in their own lives. As such, research that affects children’s lives should involve their input and participation (Langhout & Thomas, 2010).

The present study investigates student and teacher perceptions about safety during a Canadian neighborhood’s redevelopment (revitalization) and the redesign of its local school. This research is part of a larger project that investigates how neighborhood redevelopment and school redesign influence student academic achievement and wellbeing. The present study focuses on student and teacher data from 2013 and 2014, in which school and neighborhood safety emerged as a salient topic. This adds to the literature on neighborhood revitalization and mixed-income communities by offering students and teachers an opportunity to voice their opinions about safety and security in the context of school and neighborhood change. The research helps us to clarify how social bonds and interactions can be altered as a result of a neighborhood redevelopment and students’ and teachers’ experiences with such changes. Finally, this study helps to improve our understanding of whether or not the process of neighborhood change, including mixed-income neighborhood redevelopment, actually contributes to the quality of life of longstanding residents, and in particular children, who have typically little or no say about the fate of their neighborhood.

## Method

This qualitative study investigated student and teacher perceptions of safety while living or working in a neighborhood undergoing revitalization. The study and its activities were approved by the Research Ethics Board at Ryerson University and the External Research Review Committee of the Toronto District School Board. Focus groups were conducted with students and teachers at two school sites. Focus group audio recordings were transcribed and analyzed with thematic coding with a grounded theory approach (Charmaz & Bryant, 2011). All data were collected in spring-summer of 2013 and 2014.

### Participants

Focus groups were conducted with 148 students (2013 and 2014) and 45 teachers (2013), at two school sites located in the same inner city neighborhood. Participating students were 4 to 13 years old, in Kindergarten to Grade 8. Junior and Senior Kindergarten (JK/SK), for 4- and 5-year olds respectively, are part of the public education system in Ontario and elective enrolment is nearly universal. Elementary schools in Ontario typically include Kindergarten to Grade 6 or Kindergarten to Grade 8.

At each school site, student participants were divided into focus groups based on grade level. There were three focus groups with age divisions referred to as Primary (JK/SK to Grade 2), Junior (Grade 3 to Grade 6) and Intermediate (Grade 7 to Grade 8). A total of 25 student focus groups and 8 teacher focus groups were conducted, with separate focus groups for students and teachers. A total of 25 student focus groups with 148 child participants ages 4 to 13, and 8 teacher focus groups with 45 teacher participants were conducted.

### School Sites

In 2011 and 2012, a public school (RS) located in a socioeconomically disadvantaged neighborhood in downtown Toronto was closed to undergo a school redesign and rebuild. During the school redesign, which included changes to the built environment and the addition of an attached community center, students and teachers were relocated to two “feeder schools” (FS1 and FS2) in the same neighborhood. RS students in Grade 6 to Grade 8 moved to FS1, a Kindergarten to Grade 8 school. RS students from Kindergarten to Grade 5 moved to FS2, a Kindergarten to Grade 6 school. Some RS staff moved to FS1 and others moved to FS2. The RS students and staff remained at FS1 and FS2 until March of 2013. At this time, FS2 was closed, and students either went to FS1, or to RS. Teachers dispersed similarly, with some going on to work in other schools.

Throughout the school redesign process, a mixed-income initiative had been ongoing in the neighborhood since 2003, wherein old social housing units were being demolished and upgraded. Prior to redevelopment, the neighborhood was designed in the mid-20th century with low-rise buildings surrounded by park spaces and walkways, with no through streets for traffic (Dunn, 2012). Redevelopment involves the staged demolition and construction of new public housing as well as new private sector housing to create a socially mixed, denser community. The redeveloped neighborhood includes a mix of high-rise condominium buildings and houses, with new roads through the community for traffic. The public housing residents, who have the right of return, are relocated in phased waves to other buildings within the neighborhood, nearby, or to other areas of the city (Johnson, 2010).

### Data Collection and Analyses

All data in the present study were gathered through focus groups carried out at the Redesign School and Feeder School 1 in the Spring-Summer of 2013 and 2014. A semi-structured focus group method was selected because it allows facilitators to have a predetermined focus, while allowing participants to freely draw on personal and shared experiences. This allowed for a rich discussion between participants, building on one another’s comments (Kvale, 2008). With a predetermined set of initial questions, a facilitator and a research assistant were present in all focus groups. The facilitator asked questions and if needed, clarified the meaning of the questions being asked and prompted participants to engage in the discussion. The research assistants observed participants, noting non-verbal cues. Students and teachers were asked questions relating to their general feelings about the school and their experiences of relocation to a new school, neighborhood revitalization, along with their perceptions of neighborhood and school safety, among other topics.

Research team members reviewed verbatim transcriptions of audio recordings from focus groups and used inductive thematic analysis, informed by a grounded theory method (Birks & Mills, 2011) to categorize segments of student and teacher responses regarding safety within the context of neighborhood change. Grounded theory is a qualitative research approach that aims to generate theories from the data, where researchers systematically and iteratively analyze the data to identify emerging themes and theories (Denscombe, 2010). After preliminary inter-rater coding and discussion of discrepancies as they arose during meetings of the research assistants and Primary Investigator, a coding guide for data analysis was finalized, and data were coded thematically in NVivo 10.

## Results

### Violence and Safety in Schools

While the majority of students did not report feeling unsafe on the premises of their school, most students did have something to say about safety within the school and/or in the community that related to ongoing neighborhood change.

At RS, both returning and new students generally felt that the school provided them with an enhanced sense of security, in comparison to their feelings of safety within the community at large. For instance, returned RS students felt that the new, better security cameras ensure a feeling of security. One student said that there are less violent incidents since the redevelopment began, but that violent activities still occur. Other students noted that they feel safe in RS because they “have friends that back [them] up” and because RS has “good security” (Intermediate student [IS], 2013). When discussing what characteristics of RS make them feel safe, students specified: alarms, emergency lights, sprinklers, smoke detectors, and easy places to hide if there is a lockdown, such as a classroom, a locker, or a washroom. When asked if they felt safe at school, one student responded: “Ya, there is cameras, there is security guards” (Primary student [PS], 2013). Students noted their previous schools (FS1 and FS2) were different because there was “no place to hide” during a lockdown, fewer exits, and the sprinklers were “rusty and old” (IS, 2013). The majority of students reported feeling safe in the gym, classrooms, and closets.

Some students said fewer fights occur at RS than at schools they had attended previously, such as FS2, and they felt safer due to the new equipment and play areas. One student explained: “I think it’s more safe [at RS] . . . Yeah there’s not, like, gun shots and stuff.” (Junior student [JS], 2014). One student said the teachers at RS played a key role in school safety: “I feel safe because the teachers and the principal always reassure us and tell us that we are safe here and they make us feel safe” (IS, 2014). Teachers at RS reported concerns about student safety tied to ongoing construction in and around the school.

Despite those who reported a higher level of safety within RS, a large number of students reported that the level of safety decreased in RS, partly due to perceived greater permeability of the building and grounds, and partly due to the violent behavior of some individuals in the neighborhood. RS students were concerned that people could see into the school through the windows, doors were too easy to get into, playgrounds had no lockable gates, and the building construction adjacent to the school was ongoing. One student at RS remarked on the glass windows: “Five layers of glass. Glass all over. . . . in the windows. . . . Because, like, if there’s a bad guy, maybe people could break through” (JS, 2014). Similar feelings of trepidation were shared by others at FS1 who reported an incident where a window was broken at the school by someone trying to break in, and a story about a person with a knife stabbing the school window (JS, 2014). These feelings about the school’s perceived permeability were echoed by some RS teachers. Teachers at RS described the library, which is built from large glass panels, as being “almost too exposed” (Teacher, 2013). Some teachers expressed concern that in the event of a lockdown, “there is nowhere you could put kids where they wouldn’t be seen” (Teacher, 2013), and some noted that as a result, they advocated to have blinds installed and some windows frosted. Teachers noted that the later installment of blinds generally helped to increase feelings for protection. Other students said that because there were a lot of “bad people” (JS, 2014) in the neighborhood, they did not feel safe in the school or in the neighborhood.

At FS1, students had similar mixed perspectives on safety. Many students reported feeling safe due to the presence of teachers, security cameras, and the physical security of the school. Students reported that they felt safe in places like the classroom and the office. One student at FS1 explained, “I feel safe at my house, but I’m so, so safe at the school. I don’t feel safe outside” (JS, 2014). At FS1 where the school library does not have large panel windows, one student shared, “I feel safe in the library because of the shelves. You can hide back there and go ‘I’m safe’” (PS, 2014). Some students, however, said that they did not feel safe in other areas of FS1, including the washrooms, the staircases, and near large windows. They described bullying, lockdowns, and shootings nearby, as reasons for their feelings of lack of safety.

Overall, aspects of the newly redesigned school environment seemed to play a role in students’ feelings of safety (e.g., security cameras, new equipment, and play areas) and lack of safety at RS (e.g., large glass panel windows), with mention of concerns regarding neighborhood violence and school lockdowns at both FS1 and RS, and speculation from teachers regarding the role of neighborhood construction tied to redevelopment in student safety.

### Bullying in the School Setting

Given the unique circumstances relating to the ongoing construction work and changing structure of the neighborhood, including disruption of place-based familiarity and social bonds, new relationships and situations evolved that may have fostered bullying and victimization. For instance, during the move to FS2, one student remarked on the frequency of fights and heard comments that were rejecting and perhaps intimidating:

There’s just so much fights [at FS2] and some of the people . . . like the first time I went there, some of the people I knew for a long time, but some of them when I went there, they all looked at me, like, ‘Why are you coming here?’ and all that, ‘Go back to your other school. . . . We don’t need you here.’ And ‘Oh, since the RS kids came here it’s all like been so boring, it’s not alive anymore.’ And that made us feel like, sad (JS, 2013).

Students also referred to the role of adults, including the principal and their teachers, in alleviating bullying incidents. For instance, one FS1 student noted,

Every single little thing you say will, like, get you in a fight . . . Like in our class, like, there are all these bullies. Like it wasn’t really pocket knives, but it was those knives that you cut wires for or something like that. They brought it. . . and they came, two of them. One of them was purple and one of them was pink and the teacher had to take it (JS, 2014).

Another FS1 student felt teachers were intimidated themselves and had become acculturated to a certain amount of inter-student bullying:

The teachers they don’t know who to turn to for help. Sometimes the teachers they get scared because the other kid threatened them [by saying]: ‘If you say this or this I’m going to hurt you’. . . . The teachers just got used to that whole thing – like, [in] today’s age and day, teachers got used to kids being shoved around (JS, 2014).

Some of the bullying was racist or based on students’ religious beliefs. For instance, one student at RS noted, “Before this year in the other school, a kid he was making fun of [Islam]. We were, like, ‘Why are you going to do that, we never made fun of your religion’” (JS, 2013).

Bullying was seen to be a pervasive problem amongst students in all the neighborhood schools. Many students complained about incidents of bullying that they experienced or observed, including some primary students at RS who indicated that there were “lots” (PS, 2014) of bullies at the school, and that many of the bullies were in older grades. Most students agreed that involving adults (e.g., educators) was a good way to combat different forms of bullying, despite the general perception among students that educators make little difference in reducing the prevalence of bullying. Although teachers did not explicitly discuss bullying in the school setting when they retrospectively spoke about the transitional period, teachers did report a “rivalry” between students during the transition. One teacher acknowledged that there was tension between the two student bodies and said that students would “label” one another as being RS or FS students.

### Fights in the School Setting

Fights in the school setting were reported by a number of students. One student said that fights often broke out on the basketball court at FS2, with another student from RS stating, “[On the basketball court] I was kind of afraid because I saw more fights over there and people were doing dangerous things” (JS, 2013). One student remarked on the level of ‘strictness’ of school administrators, connecting ‘stricter’ administrators with fewer school fights, and sharing perceptions of differences across schools. Bullying can instigate or escalate to physical altercations. One student talked about being bullied by FS2 students and describing a fight that broke out as a result. Another student at FS1 said that fights were common in the neighborhood along with other types of violence, and that: “Kids are getting hurt and stuff—every day,” with some children “ganging up” against one another (JS, 2013). The neighborhood schools implemented programing to help remedy issues tied to violence. For example, one student at FS1 referred to the existence of anger management classes, and other students revealed, “I’m in one of those classes” (JS, 2013). Similar to situations of bullying, students had mixed perspectives about whether educators were effective in preventing or stopping fights. For example, one student described teachers as, “just, like, standing there and watching,” (JS, 2014) while other students suggested that having more teachers attempt to deescalate situations was helpful. A teacher discussed the transition into RS (2013) in relation to behavioral incidents saying,

I really felt when I first came to this school – and maybe it’s me becoming better at managing my position – but I really felt like it was a lot more sense of urgency all the time, a sense of scrambling, like there were always incidents. There were big incidents. There were big fights, things like that. . .the behavior piece was a big part of your daily job. I feel like that has really improved, like hugely. I believe our number of suspensions is down. I know that the number of office referrals is way down.

### Gang Activity in the Community

A number of junior and intermediate students in both FS1 and RS spoke about gang activity in the community. Students’ perceptions of gang activity in the neighborhood were mixed, where some felt the presence of gangs had diminished, and others felt gangs in the neighborhood were active. The students who felt that gang activity had diminished explained that “[there] used to be gangs really bad” (JS, 2014), with suggestions that a lot of the gang members had died. Referring to the neighborhood’s impact on the school climate, some students suggested that there were fewer gangs in the area near the school, but others disputed this claim. Some students said that gangs were still active in the neighborhood, that they knew of individuals still involved in a gang, and that some gang-related activities had simply relocated. Other students mentioned school lockdowns, gang-related behaviors and criminal activities in the area as reasons for feeling unsafe in school.

When asked whether the redevelopment efforts (which include new youth projects) were changing gang activity in the neighborhood, one student was adamant, saying, “No. They might be changing [the neighborhood], but they are not going to change the people that are here” (JS, 2014). One educator (2013) suggested that since the revitalization, “young kids are getting into things at much earlier ages whether its weapons, robbing, stealing,” as gangs have become “disjointed” and “everyone is kind of vying for different positions.” Other teachers, were more hopeful, pointing to new employment opportunities due to neighborhood redevelopment, because “that’s a big issue for kids, choosing to work versus gangs and drugs”.

However, some students were skeptical that all forms of violence occurring in the neighborhood were gang-related. One student suggested that police officers’ investigations may be based on stereotypes and the quick assumption that some forms of violence are gang-related. The student described a specific situation where someone died in one of the buildings in the neighborhood, saying, “There’s somebody that passed away in one of the buildings, and they were like, ‘Oh it was gang-related’, [but] it wasn’t at all. They just thought it was because of how he looked and everything, his hair and this and this and that” (IS, 2013). In response to this, another student said, “it’s not fair” (IS, 2013). Gang activity in the community was a salient topic of discussion among students and teachers, with skepticism from some students that redevelopment reduced gang activity, and others alluding to unfair assumptions of gang activity due to profiling.

### Territory and Rivalry in the Neighborhood

Some junior and intermediate students remarked on issues of territory in the neighborhood and rivalry between schools, with the different groups of students coming together in RS and FS1 due to school redesign. One student referred to the turf-based rivalry that exists between different schools and how that initially prejudiced her feelings about moving to RS:

When I moved here [to RS] I was mad because, well, like the reputation they had.. . . They [former school friends] don’t like this school. They told me so much different things about it, but when I came here it was so different, and, like, here I am the same person I was back then . . . ‘cause I have the same personality, I haven’t changed (IS, 2014).

Because of what she had been told, she was angry, upset, and wary during her initial time at RS, but reported that she quickly got over that and then liked the school.

Teachers also commented about rivalries across the different areas of the neighborhood. One teacher noted, “Rivalries between north and south [areas of the original social housing development] were very real” (Teacher, 2013). However, the social and spatial dynamics have shifted with the influx of students in the newly redesigned RS. Another teacher at RS (2013) noted,

I am incredibly surprised about how well it has gone. One of the big changes that we were worried about when we moved out was this whole idea, this notion of south and north. . . and that you don’t cross the lines. But the kids really did surprise me, which goes to show sometimes we have these perceptions. That we worry about them and in fact it is fine.

### Neighborhood Crime

A number of students at both schools believed that the rate of crime in the neighborhood had not been reduced as a result of revitalization. In particular, they were upset that shootings still took place, saying that shootings happen “every other month” (JS, 2013). Another student reported, “I saw on TV that somebody [shot] somebody in the car and the car crashed” (PS, 2014). They also referred to the lockdowns that have happened at RS, as well as the people in the neighborhood who yell at children. They noted the various places in the neighborhood where people have died from violence. One student talked about shootings starting up in the neighborhood again: “Before there were lots of shootings and then they stopped for a bit, now they are back” (JS, 2013).

Educators also remarked on the extent of criminal activities and vandalism within the neighborhood. For example, one educator (2013, RS) said:

We have circle time [with students] – you can call it whatever you want, but we basically talk about how our weekend went and whatever is on their mind. . . such as somebody not wanting to leave their apartment because somebody is dealing drugs outside, or they are just not allowed to go outside because there is nothing for them to do there. One of the parks they go to. . . there is often people with mental health issues and [addiction], so a lot of parents and guardians choose to not let their child have some freedom in that sense.

### Community Safety

Junior and intermediate students speculated about safety in the broader community. When asked whether violence in the neighborhood had changed as a result of the revitalization process, one student said, “It hasn’t changed,” but the student noted that media reports on violence no longer “say [the name of the neighborhood] because they want people to move in” (IS, 2013); instead, the address or the name of the building is often provided. Another student agreed that the level of violence in the neighborhood has remained the same since neighborhood redevelopment began. Some students noted, “the roads are open [due to redevelopment] for the police cars to go in or out. . . but they’re still not catching anyone” (IS, 2013). Other students noted that the level of violence has been “better since 2005. . . but it’s not gone” (IS, 2013).

One student (2013) suggested that the relocation and introduction of new people due to redevelopment would instigate conflict between original and new residents, suggesting class enmity, with incoming residents perhaps looking down upon longstanding residents:

There’s going to be more violence happening. . . You’re bringing in people from different neighborhoods against us. We’re not saying that we are harmful to them. They must feel offended by us and try to do something to us that’s causing more violence.

The forced relocation of residents during neighborhood redevelopment construction concerned some students. Remarking on safety concerns, one student noted, “[it] doesn’t make sense,” because it will create “more violence in another area” (IS, 2013). The significance of territory was raised again when a student noted, “they may shoot you or something” because you cannot mix neighborhoods, and when people know you are from this neighborhood, they will say, “I don’t like you” (IS, 2013). Another student suggested that you cannot just “throw” this neighborhood’s residents into a new community, but rather, the move should be done slowly.

The ongoing construction work itself raised concerns about safety among students. For example, an intermediate student (2013) said that neighborhood redevelopment is “dangerous,” noting that a playground was closed because of safety concerns due to construction. Students reported windows being smashed with rocks in empty buildings slated for demolition, that there is increased air pollution due to construction, and that the community is “dirty” and “gross” (IS, 2013). Some new buildings, students explained, have a pass key to enter while others have a front desk with security staff where visitors must check in. However, many students noted that the security in their own building is low, with one student saying that his/her building has a security officer, but he is rarely ever seen. Students shared their concerns about safety in the broader community, discussing their perspectives on the stability neighborhood violence, social mix, describing neighborhood construction, and building security.

## Discussion

Students and teachers both attested to the multifaceted nature of violence and safety issues in the different areas of the neighborhood, especially within the spatial boundaries of public schools. Students were generally skeptical about the positive effects of neighborhood revitalization on safety, citing the possible relocation of violence to different areas of the city and potential rivalry between longstanding and new residents in the neighborhood. Students even reported newer forms of violence that may not have been present prior to neighborhood change, including clashes between longstanding and incoming students at feeder schools, and vandalism tied to construction. Student and teacher perceptions of safety within the context of neighborhood revitalization were generally consistent with the literature on the effects of neighborhood change initiatives on violence. Specifically, students were pessimistic about reduction in violence (August, 2014), and the ability of social mixing to address the root causes of violence in the neighborhood (Lipman, 2008). Students recognized that some of the ongoing neighborhood revitalization efforts aimed to reduce violence and crime in the community, such as creating new roads so that police officers can more easily patrol the neighborhood, had not yet reduced the level of crime. This aligns with previous research that has challenged the “broken windows” theory (Wilson & Kelling, 1982).

Sampson and Raudenbush (1999) have suggested that crime and violence is more strongly related to other neighborhood level factors, such as neighborhood collective efficacy, rather than disorderly conditions. In the present study, social cohesion seemed to influence community violence, where students spoke about the potential for violence to occur as new divides and rivalries could be developed when longstanding residents were relocated and as new residents move into the neighborhood undergoing redevelopment. Students also discussed how community violence negatively influenced their feelings of safety within school. This is congruent with numerous studies that have found that witnessing or hearing about community violence impacts children’s emotional regulation, traumatization and fear for safety (Fowler et al., 2009).

Students, however, felt safer in school than in certain areas of the neighborhood, partly due to the installment of new security measures. This does not correspond with the findings of Perumean-Chaney and Sutton (2013), who report the negative effects of visible security measures on students’ perceptions of safety. This difference in findings may be rooted in neighborhood redevelopment and the resultant school relocation of many students from one school to another (e.g., Thompson et al., 2013); this neighborhood and school change was not present in Perumean-Chaney and Sutton’s study. The school relocation process which, according to many students in the present study, triggered bullying and physical fights over the clashing of different student populations, caused further concern for safety. Though students’ perceptions of visible security measures reassured them of their safety, a more prominent indicator of perceived safety was the presence of social ties. As Clampet-Lundquist (2010) and Thompson et al. (2013) suggest, perceived risk of victimization increases with relocation, substantiating the importance of social networks in communities that are prone to high rates of violence.

The prominence of social ties as an indicator of feelings of safety and security in our findings most notably corroborate the arguments made by Kim et al. (2013), contending that a distinction between the concepts of space and place help in the understanding of spatial factors contributing to crime, and by extension, perceptions thereof. Physical features associated with any given locale (space), argue Kim et al. (2013), do not contribute as effectively to the understandings of crime and safety as do the social and emotional ties that form in a physical setting (place). Similarly, in the present study, students’ feelings of safety in school were described as being connected to the presence of supportive and encouraging teachers and school administrators whom the students turned to in times of distress. Although some students perceived teachers as unable to intervene and resolve incidents of bullying, most students agreed that involving adults is an effective way to combat different forms of bullying and violence taking place in school (e.g., Cortes & Kochenderfer-Ladd, 2014). Bullying was a much more salient topic for students, in comparison to teachers, who instead discussed physical fights in the school setting.

The transitional relocation of students to feeder schools during the redesign of RS resulted in some students feeling unwanted and unsafe due to alienating remarks made by feeder school students. Students’ ability to trust one another and to form new relationships may be negatively impacted by their continuous exposure to violence in the community (Overstreet, 2000). However, direct exposure might not be the only way that students’ ability to form relationships is affected. As noted by one teacher, many parents do not allow children to play outside due to potential violence that can take place in play areas such as public parks in the neighborhood. Such protective measures may stem from parental beliefs about how neighborhood mechanisms can affect their children negatively. However, these beliefs can deprive children of the ability to form relationships outside of the school context and even alter the quality of social networks (Galster & Santiago, 2006). For many students, the very existence of violence in the neighborhood was perceived to be associated with feeling unsafe inside the physical boundaries of school. The level of neighborhood crime can predict the extent to which students perceive their school to be safe (McCoy et al., 2013). With stated fears about intruders and the perceived ineffectiveness of school windows to keep students safe from the violence in the community, both students and teachers revealed that aspects of the school redesign initiative might not actually help to instill feelings of safety within the physical boundaries of school in this neighborhood. However, in general, many students regarded their school as a safe haven, away from criminal activities in the neighborhood.

Both students and teachers were acutely aware of the occurrence of neighborhood crime, with many perceiving the prevalence of criminal activities to have remained steady even after changes were introduced in the neighborhood. As suggested by August (2014), new venues for conflict and violence can be created by the introduction of “socially mixed” neighborhoods in which the socioeconomic, ethnic, and cultural characteristics of longstanding and incoming students are highly diverse. In fact, when asked about the impact of relocation and revitalization, students expressed concern about the “dangers” of mixing people from different neighborhoods, as socio-cultural clashing seemed a plausible outcome, both for newly incoming residents and those moving from the neighborhood to other areas. Students were skeptical about the formation of meaningful social ties between longstanding and new residents (Clampet-Lundquist, 2010; Tach, 2009), bringing up the notion of stigma as a reason for this feeling. This raises questions regarding the notion of “place destigmatization” as a positive outcome for public housing neighborhoods undergoing mixed-income revitalization and merits further study (e.g., Dunn, 2012). Students indicated that not only are they aware of the stigma of living in this particular neighborhood, but that this stigma plays a central role in their interactions with others and implied that it may fuel violence in the neighborhood.

Students also noted that they felt the new residents would look down upon longstanding residents and could sense that new residents were “offended” by longstanding residents’ presence. Students commented on how the media reported building addresses rather than using their neighborhood name, demonstrating an effort to develop a new post-redevelopment neighborhood identity. Hwang (2016) highlights how residents’ constructions of neighborhood identity and boundaries varyin the context of gentrifying neighborhoods, where new residents’ constructions can alienate longstanding marginalized residents.

Students noted that the underlying causes of violence in the neighborhood are not addressed by neighborhood redevelopment (e.g., Lipman, 2008). For example, in reference to gang activities, one student shared that neighborhood revitalization does not actually change the characteristics of residents, and hence in their view, does not help make the neighborhood safer, and that gang activities cannot be fixed simply by new buildings and residents. In line with August’s (2014) arguments, students and teachers were also skeptical about a reduction in the rate of gang activities in the neighborhood, instead suggesting that gang members may have relocated within the same neighborhood. Furthermore, some students noted that much of what is perceived as gang-related may not be so; instead, that it is rooted in racial stereotypes held about certain residents of this neighborhood. Racial stereotyping was also reported within schools, resulting in bullying among students during relocation. Although racial integration may be an intended outcome of neighborhood revitalization (Kost, 2012), it is not clear from the present study as to whether such initiatives actually challenge marginalization due to race, ethnicity, culture, or language (Lipman, 2009). Social integration itself requires the formation of close interpersonal ties, something that is generally absent between longstanding and incoming residents (Dunn, 2012; Tach, 2009).

## Conclusion

Including children’s voices is a necessary step when planning, implementing, and evaluating policy initiatives that directly affect their lives. This study describes student and teacher perspectives on safety in an inner city and marginalized neighborhood undergoing revitalization, alongside the redesign of a local school in the same neighborhood. The displacement of families and students, tied to housing relocation and student school mobility created increased vulnerability of students to bullying, safety concerns at school due to construction and the design features of glass panels, as well as the displacement of place-based familiarity and social bonds. Despite this, the majority of students felt safe on the premises of the school and noticed the security measures in their school surroundings. Furthermore, junior and intermediate students were acutely aware of community level violence, noting gaps in building security, criminal and gang activity in the neighborhood, along with perspectives on racial stereotyping, and the mixing of longstanding and new residents in the neighborhood. Students were generally skeptical that neighborhood redevelopment would address the root causes of violence. Given the strong empirical support for the role of community safety in students’ psychological well-being and educational achievement, the findings highlight the importance of community ties in helping to foster feelings of safety and security within the context of neighborhood change and have implications for programing, practice, and policy.
